# Public Unawareness of Renal Function: A Questionnaire Survey at a Health Promotion Seminar

**DOI:** 10.3390/jcm14030664

**Published:** 2025-01-21

**Authors:** Yukinori Aimiya, Sho Hasegawa, Mikio Sakakibara, Midori Hasegawa, Naotake Tsuboi, Naoki Nakagawa, Shigeki Yamada

**Affiliations:** 1 Sugi Pharmacy Co., Ltd., Obu 474-0011, Japan; m-sakakibara@sugi-pharmacy.co.jp; 2 Department of Pharmacotherapeutics and Informatics, Fujita Health University School of Medicine, Toyoake 470-1192, Japan; sho.hasegawa@fujita-hu.ac.jp (S.H.); syamada@fujita-hu.ac.jp (S.Y.); 3 Department of Nephrology, Fujita Health University School of Medicine, Toyoake 470-1192, Japan; mhase@fujita-hu.ac.jp (M.H.); nao-take@fujita-hu.ac.jp (N.T.); 4 Division of Cardiology and Nephrology, Department of Internal Medicine, Asahikawa Medical University, Asahikawa 078-8510, Japan; naka-nao@asahikawa-med.ac.jp

**Keywords:** chronic kidney disease, renal function, questionnaire survey, pharmacy, education

## Abstract

**Background:** Progression of chronic kidney disease (CKD) increases the risk of complications such as cardiovascular disease; however, knowledge regarding renal function in the general population is low. We aimed to determine factors necessitating CKD education in the general population. **Methods:** Participants for a health promotion seminar were recruited via the Sugiura Memorial Foundation website and Sugi Pharmacy stores. Those who agreed to participate in the seminar were included in the questionnaire survey after a health seminar. **Results:** Out of 1548 participants, 1050 answered all questionnaire items, resulting in a valid response rate of 67.83%. Multivariable analysis revealed that sex (OR = 0.611), pharmacy consultations (OR = 0.661), receiving a blood test within 1 year (OR = 0.268), awareness of blood pressure (OR = 0.038), and knowledge of blood glucose level (OR = 0.099) were factors for unawareness of renal function. **Conclusions:** This study suggests that female individuals unaware of their blood pressure or glucose levels, those who have not had a blood test within 1 year, and those who have not sought health consultations need education on renal function.

## 1. Introduction

The global prevalence of chronic kidney disease (CKD) is increasing, and epidemiological trends pose a serious clinical challenge. CKD progression increases the risk of complications, such as cardiovascular disease, often necessitating dialysis or kidney transplantation. Japan has one of the highest incidences of end-stage kidney disease (ESKD). Although the number of patients with ESKD requiring renal replacement therapy has remained stable or decreased in recent years, owing to Japan’s aging population, this number is expected to increase in the future [1].

To prevent renal function decline, patients should monitor and control blood pressure and blood sugar levels; however, achieving this requires lifestyle changes and better adherence to oral medications for patients with chronic diseases. Health literacy is associated with medication adherence in chronic diseases [2] and consistently correlates with good self-management behaviors in individuals with or at risk of CKD [3]. However, CKD awareness among individuals with an estimated glomerular filtration rate (eGFR) < 60 mL/min/1.73 m^2^ was 26.5%, according to a systematic review and meta-analysis of 32 studies [4]. In Japan, general knowledge and information about renal function are inadequate [5]. Chronic diseases such as diabetes, hypertension, and dyslipidemia are often followed up on at local pharmacies, providing ample opportunities for pharmacists to share blood pressure and hemoglobin A1_c_ measurement results with patients [6]. However, many community pharmacies lack awareness of renal function [7]. Patient and healthcare worker education is crucial to addressing this issue. Although structured group education [8,9] has demonstrated effectiveness in the care of patients with CKD, there are methodological limitations and a high heterogeneity of interventions and outcomes [10]. Existing health literacy indicators lack specificity for assessing CKD literacy, and disease-specific and detailed indicators are desirable. The health literacy measurement tools do not adequately evaluate CKD literacy; thus, tools for typical diseases are ideal.

Kidney Disease: Improving Global Outcomes (KDIGO) and the National Health and Nutrition Examination Survey (NHANES) in the US have previously been used to assess CKD awareness [4]. However, no simple questionnaire items have been developed for the general population. To the best of our knowledge, no scales are available to evaluate CKD literacy. Creating a scale requires considering factors such as applicability to the general population, the number of items, and avoiding redundancy. Furthermore, factors necessitating CKD education in the general population have not been determined. We conducted a survey during the seminar featuring a lecture on polypharmacy in patients with renal failure to evaluate the availability of our questionnaire. The participants with or without CKD were considered to have a certain level of health literacy and intelligence because participation in the seminar was voluntary. Collecting data from a population with these characteristics provides a basis for evaluating the validity of the questionnaire.

This study aimed to propose a simple questionnaire item and identify factors affecting the need for CKD education in the general population.

## 2. Materials and Methods

### 2.1. Data Source and Study Design

The participants of the health promotion seminar regarding polypharmacy in patients with CKD were recruited via the Sugiura Memorial Foundation website and Sugi Pharmacy stores. Those who attended the health promotion seminar held by the Sugiura Memorial Foundation on 10 September 2023 and agreed to participate were included in the survey. The health promotion seminar was held in Aichi Prefecture, and participants from other prefectures (Saitama, Tokyo, Osaka, Kyoto, and Ishikawa) joined via webinar. The health promotion seminar featured a lecture on polypharmacy in patients with renal failure. After the seminar, a survey was conducted to accommodate varying levels of digital literacy in participants, using either paper-based questionnaires or Google Forms. The questionnaire contents were as follows:

1. Age (<65 years old or not)
2. Sex (female or male)
3. Have you ever sought health advice at a pharmacy?
4. Have you ever had a blood test in the past year?
5. Do you know your renal function?
6. Do you know your blood pressure level?
7. Do you know your blood glucose level?
8. Are you aware of salt reduction efforts?
9. Are you aware of your protein intake?
10. Do you consume two or more fruits or vegetables daily?
11. Do you consume five or more glasses of fluids (water, juice, coffee, tea, and milk) daily?
12. Do you take dietary supplements daily?
13. Do you use any medications?
14. Do you have a family pharmacy?
15. Do you maintain a regular exercise routine (30 min, at least twice a week for over a year)?

The participants aged ≥18 years who answered, “I know my renal function” and “I do not know my renal function” were divided into the “knowing renal function” and “not knowing renal function” groups, respectively. We compared the questionnaire results between the two groups. All data were anonymized.

### 2.2. Statistical Analyses

Categorical variables are presented as numbers and percentages. To evaluate the association between renal function unawareness and questionnaire results, a chi-square test was used to compare categorical variables. Multivariate analyses were conducted to assess the above association as the independent variable and evaluated the goodness of fit by using the Hosmer–Lemeshow statistical test. Multicollinearity was assessed using the variance inflation factor (VIF). Statistical significance was defined as *p* < 0.05. All statistical analyses were performed using Statistical Package for the Social Sciences (version 24.0; SPSS Inc., Chicago, IL, USA). The results of statistical analyses were validated by two researchers.

### 2.3. Ethical Approval

This study was approved by the ethics board of Sugi Holdings (approval number: 2023-009, approval date: 18 August 2023). The survey was conducted ethically, ensuring the proper handling of personal information and the voluntary participation of participants in the survey. Google Docs and Google Forms were used to obtain consent from the participants.

## 3. Results

### 3.1. Participant Characteristics

Participant characteristics are shown in Table 1. Out of 1548 participants, 1050 (paper sheets: *n* = 637, Google Forms: *n* = 413) answered all questionnaire items, resulting in a valid response rate of 67.83%. Characteristics of the two groups are presented in Table 1. Approximately 80% of participants unaware of their kidney function were aware of their blood pressure levels. In contrast, approximately 70% of participants unaware of their kidney function were unaware of their blood sugar levels.

A chi-square test indicated that participants who were <65 years and female participants were significantly more likely to be unaware of their renal function. Additionally, never consulting about health at a pharmacy (*p* < 0.001), never having recent blood tests (*p* < 0.001), unawareness of blood pressure (*p* < 0.001) and glucose levels (*p* < 0.001), non-use of medication (*p* < 0.001), not having a family pharmacy (*p* = 0.001), and no exercise habits (*p* < 0.001) were all associated with not knowing one’s renal function. Awareness of protein, salt, fruit, type of fluid, and dietary supplement intake was not associated with unawareness of renal function.

### 3.2. Results of Multivariable Analysis

The results of multivariable analysis are shown in Table 2. Multivariable analysis revealed that age, awareness of salt intake, protein intake, fruit or vegetable intake, fluid intake, dietary supplement intake, medication use, family pharmacy, and exercise habits did not affect unawareness of renal function. In contrast, male gender (OR = 0.611, *p* = 0.007), receiving a health consultation at a pharmacy (OR = 0.661, *p* = 0.026), receiving a blood test within one year (OR = 0.268, *p* = 0.010), and knowledge of blood pressure (OR = 0.038, *p* = 0.001) and blood glucose (OR = 0.099, *p* < 0.001) were associated with significantly reduced ORs of unawareness. The model exhibited good fitness according to the Hosmer–Lemeshow test (*p* = 0.317). The VIFs for renal function unawareness were 1.004 (sex), 1.020 (health advice at a pharmacy), 1.094 (receiving a blood test within 1 year), 1.135 (knowledge of blood pressure), and 1.125 (blood glucose levels); thus, multicollinearity was not detected in this model.

## 4. Discussion

Some studies have shown that patients’ awareness of CKD varies depending on how questions are phrased [4,11]. In this study, we targeted a general population that included healthy individuals. Therefore, the questionnaire used the term “renal function” rather than specific indicators such as eGFR or creatinine levels to assess awareness of renal function in everyday contexts. The valid response rate for our questionnaire was >65%; therefore, a simple questionnaire item might contribute to an increased response rate. Additionally, this study revealed that around 40% of participants were aware of their own kidney function. Given that many participants might be more motivated to prioritize their health, the frequency of renal function awareness was the same degree in previous meta-analyses on CKD awareness [4]. GFR values were not obtained from participants in our survey; therefore, the frequency of participants with eGFR < 60 mL/min/1.73 m^2^ remains unknown. However, the results suggest that awareness of renal function in the general population in Japan is still insufficient.

The univariable analysis identified under 65 years old as a factor associated with kidney function unawareness. As renal function typically declines with age, participants aged ≥65 years may have had more opportunities for education regarding kidney disease from their physicians and medical staff than the young participants. Given that CKD progresses slowly and symptoms may not manifest, young people often lack the opportunity to notice changes in their renal function. In Japan, a nationwide annual health checkup program has been implemented since 2008. Health checkups and notifications regarding one’s health are crucial in preventing CKD progression. Medical staff should emphasize the importance of young people knowing their own renal function [12].

Sex differences affected renal function unawareness in our survey. In Japan, a previous study suggested that males have high adherence to health guidance invitations through a nationwide annual health checkup program [13]. The proportion of men with access to the annual health checkup program was higher than that of females because of the high employment rate. The number of pharmacists is higher in Japan than that in other countries [14]. Pharmacies could play a valuable role in educating females who do not have access to the annual health checkup program.

A close relationship was observed between awareness of blood pressure, blood sugar levels, and renal function. Hypertension and diabetes are known to contribute to CKD progression, and patient education often includes health information related to these conditions. However, approximately 80% of participants unaware of their kidney function were aware of their blood pressure levels. Although blood pressure control is an important factor in preventing renal function decline, our results suggest that most people are unaware of the relationship between blood pressure and renal function. In contrast, awareness of blood sugar levels was only approximately 30% among participants unaware of their kidney function, which was significantly lower than that of participants who were aware of their kidney function. Given that most participants knew the importance of hypertension in their health, improving awareness of renal function requires education that focuses more on the relationship between hypertension and CKD rather than on diabetes and CKD. Exercise habits and awareness of dietary habits did not directly impact unawareness of renal function. This suggests that participants may not have recognized an association between exercise habits, dietary habits, and kidney function. The Looking Action for Health in Diabetes study established dietary goals, such as a calorie intake of <30% of calories from fat and >15% from protein [15], along with exercise requirements to reduce body weight. This trial suggested that intensive lifestyle interventions reduce the incidence of high-risk CKD [16]. Although the effect of a sodium-restricted diet on preventing ESKD remains unclear, this diet decreased blood pressure and albuminuria [17]. The impact of exercise habits and dietary awareness on renal function is partial, which may reflect the negative results regarding renal function awareness.

Our survey revealed that participants who consulted about their health at a pharmacy were aware of their kidney function. Pharmacists’ participation in CKD treatment enhanced the optimization of treatment and reduced the hospitalization frequency related to drug-induced renal failure [18]. Comprehensive education regarding lifestyle, nutrition, and medications for outpatients prevents CKD progression [19]. Our results align with previous studies suggesting that patients would benefit from information focused on renal function and increased opportunities for consultation when receiving medication guidance. Although having a family pharmacy was not identified as a factor in renal function unawareness by multivariable analysis, 334 participants who had a family pharmacy were still unaware of their own kidney function. This suggests that family pharmacists may have provided insufficient information regarding renal function. Considering these results, it is necessary for pharmacists to obtain professional qualifications to enhance their education. In the future, measures should be implemented to improve renal function literacy in pharmacies based on these results. To realize a strategy to improve renal function awareness in pharmacies, the role of registered dietitians is also crucial in Japan. Therefore, pharmacies should serve as the primary access point to healthcare for residents.

In this study, the questionnaire survey was conducted after the seminar. While the seminar might have prompted participants to recall their kidney function, we believe its impact on the results is minimal. Participants without prior awareness of their kidney function would likely still face difficulty recalling it, even with the seminar. Therefore, to minimize potential bias, the questionnaire was designed to encourage objective responses, irrespective of the seminar content.

To accommodate varying levels of digital literacy, the survey used either paper-based questionnaires or Google Forms. Approximately 60% of the participants (637 of the 1050) responded using paper-based questionnaires, indicating that most individuals were not influenced by digital literacy. Furthermore, approximately 76% of the participants (795 of the 1050) were aged <65 years. According to a survey by the Ministry of Internal Affairs and Communications, approximately 90% of this age group in Japan use the Internet [20]. Therefore, it suggests that the bias due to digital literacy in this study is minimal.

This study had several limitations. First, medical data regarding underlying diseases and blood tests were not obtained, making the proportion of patients with renal failure among participants unclear. Second, the results were based on self-reported questionnaires, potentially introducing response bias. Third, this survey was conducted at a health promotion seminar hosted by the Sugiura Memorial Foundation; thus, further studies are needed to evaluate whether the present results are applicable to other facilities. Fourth, all participants in this survey were Japanese. Further studies on other populations are needed to validate the results of our survey. Fifth, a simplified questionnaire was utilized to measure health literacy to reduce selection bias. No scales were available to evaluate CKD literacy; thus, there is no other relevant survey to confirm the reliability of this result. Therefore, although our survey results could not be compared with those of previous studies, the survey achieved a valid response rate exceeding 65%. Sixth, careful consideration should be given to the possibility of selection bias affecting the survey results in this study. Participation in the survey was voluntary; thus, participants’ behavior in attending the seminar indicates a high level of health awareness. Therefore, there is a possibility that responses may be biased in a favorable direction. If such a tendency exists, there is a possible risk that the survey results may not accurately reflect the reality of patients with CKD in the general clinical setting. Seventh, it is anticipated that family members or companions may offer advice while participants complete the questionnaire in the seminar. Under such circumstances, the individual’s actual health literacy level may not be accurately assessed, and the responses may appear more positive than they really are. This risk could lead to an overestimation of the surveyed person’s literacy. Therefore, the questionnaire should be designed to be completed individually. Furthermore, we believe that the influence of others can be minimized by incorporating a mechanism that clearly states that the individual provides the answers. The seminar participants who are the target of the survey are likely to be a group with an inherent level of health literacy. Therefore, careful discussion and additional validation will be required when attempting to identify low literacy demographics based on the results obtained from this survey. To ensure the diversity of the survey population, it is important to include segments of the population that are not necessarily highly health-conscious. Specifically, conducting the survey at general clinics and health screening sites is useful for collecting data from a broad demographic.

In conclusion, the present study suggests that females, the population unaware of blood pressure and blood glucose levels, the population without blood tests within one year, and those without the experience of health consultation require education regarding renal function.

## Figures and Tables

**Table 1:** Group characteristics.

Characteristics	Not Knowing Renal Function (*n* = 669)	Knowing Renal Function (*n* = 381)	*p*-Value
Age (years)			
<65 years, *n* (%)	533 (79.7)	262 (68.8)	<0.001
≥65 years, *n* (%)	136 (20.3)	119 (31.2)	
Sex			
Male, *n* (%)	143 (21.4)	122 (32.0)	<0.001
Female, *n* (%)	526 (78.6)	259 (68.0)	
Have you ever sought health advice at a pharmacy?			
Ever, *n* (%)	130 (19.4)	122 (32.0)	<0.001
Never, *n* (%)	539 (80.6)	259 (68.0)	
Have you had a blood test in the past year?			
Yes, *n* (%)	586 (87.6)	376 (98.7)	<0.001
No, *n* (%)	83 (12.4)	5 (1.3)	
Do you know your blood pressure level?			
Yes, *n* (%)	557 (83.3)	380 (99.7)	<0.001
No, *n* (%)	112 (16.7)	1 (0.3)	
Do you know your blood glucose level?			
Yes, *n* (%)	188 (28.1)	318 (83.5)	<0.001
No, *n* (%)	481 (71.9)	63 (16.5)	
Are you aware of salt reduction efforts?			
Yes, *n* (%)	424 (63.4)	263 (69.0)	0.064
No, *n* (%)	245 (36.6)	118 (31.0)	
Are you aware of your protein intake?			
Yes, *n* (%)	645 (96.4)	375 (98.4)	0.060
No, *n* (%)	24 (3.6)	6 (1.6)	
Do you consume two or more fruits or vegetables daily?			
Yes, *n* (%)	529 (79.1)	313(82.2)	0.229
No, *n* (%)	140 (20.9)	68 (17.8)	
Do you consume five or more glasses of fluids (water, juice, coffee, tea, milk) daily?			
Yes, *n* (%)	389 (58.1)	237 (62.2)	0.198
No, *n* (%)	280 (41.9)	144 (37.8)	
Do you take dietary supplements daily?			
Yes, *n* (%)	291 (43.5)	175 (45.9)	0.445
No, *n* (%)	378 (56.5)	206 (54.1)	
Do you use any medications?			
Yes, *n* (%)	344 (51.4)	241(63.3)	<0.001
No, *n* (%)	325 (48.6)	140 (36.7)	
Do you have a family pharmacy?			
Yes, *n* (%)	334 (49.9)	232 (60.9)	0.001
No, *n* (%)	335 (50.1)	149 (39.1)	
Do you maintain a regular exercise routine (30 min, at least twice a week for over a year)?			
Yes, *n* (%)	275 (41.1)	198 (52.0)	0.001
No, *n* (%)	394 (58.9)	183 (48.0)	

**Table 2:** Factors affecting renal function unawareness.

Factors	Univariable Analysis		Multivariable Analysis	
	Odds Ratio (95%CI)	*p*-Value	Odds Ratio (95%CI)	*p*-Value
Characteristics				
Age				
<65 years	1.780 (1.336–2.372)	<0.001	1.128 (0.779–1.633)	0.523
≥65 years	Reference		Reference	
Sex				
Male	0.577 (0.435–0.766)	<0.001	0.611 (0.426–0.874)	0.007
Female	Reference		Reference	
Have you ever sought health advice at a pharmacy?				
Ever	0.512 (0.384–0.683)	<0.001	0.661 (0.459–0.951)	0.026
Never	Reference		Reference	
Have you had a blood test within the past year?				
Yes	0.094 (0.038–0.234)	<0.001	0.268 (0.098–0.733)	0.010
No	Reference		Reference	
Do you know your blood pressure level?				
Yes	0.013 (0.002–0.094)	<0.001	0.038 (0.005–0.282)	0.001
No	Reference		Reference	
Do you know your blood glucose level?				
Yes	0.077 (0.056–0.106)	<0.001	0.099 (0.071–0.139)	<0.001
No	Reference		Reference	
Are you aware of salt reduction efforts?				
Yes	0.776 (0.594–1.015)	0.064	1.397 (0.987–1.978)	0.059
No	Reference		Reference	
Are you aware of your protein intake?				
Yes	0.430 (0.174–1.061)	0.060	0.585 (0.189–1.805)	0.351
No	Reference		Reference	
Do you consume two or more fruits or vegetables daily?				
Yes	0.821 (0.595–1.132)	0.229	0.923 (0.610–1.396)	0.703
No	Reference		Reference	
Do you consume five or more glasses of fluids (water, juice, coffee, tea, milk) daily?				
Yes	0.844 (0.652–1.092)	0.198	0.896 (0.653–1.229)	0.496
No	Reference		Reference	
Do you take dietary supplements daily?				
Yes	0.906 (0.704–1.167)	0.445	1.217 (0.890–1.666)	0.219
No	Reference		Reference	
Do you use any medications?				
Yes	0.615 (0.475–0.796)	<0.001	0.914 (0.646–1.293)	0.611
No	Reference		Reference	
Do you have a family pharmacy?				
Yes	0.640 (0.496–0.827)	0.001	0.893 (0.630–1.266)	0.526
No	Reference		Reference	
Do you maintain a regular exercise routine (30 min, at least twice a week for over a year)?				
Yes	0.645 (0.501–0.831)	0.001	0.834 (0.605–1.149)	0.267
No	Reference		Reference	

## Data Availability

The original contributions presented in this study are included in the article. Further inquiries can be directed to the corresponding author.
